# Comparison of histochemical methods for the analysis of eosinophils and mast cells using a porcine model of eosinophilic esophagitis

**DOI:** 10.3389/fvets.2025.1540995

**Published:** 2025-03-19

**Authors:** Douglas B. Snider, David K. Meyerholz, Evan S. Dellon, Lizette M. Cortes, Akash Karri, Anthony T. Blikslager, Scott Laster, Tobias Käser, Glenn Cruse

**Affiliations:** ^1^Department of Molecular Biomedical Sciences, College of Veterinary Medicine, North Carolina State University, Raleigh, NC, United States; ^2^Comparative Medicine Institute, North Carolina State University, Raleigh, NC, United States; ^3^Comparative Medicine and Translational Research Training Program, North Carolina State University, Raleigh, NC, United States; ^4^Department of Pathology, University of Iowa Carver College of Medicine, Iowa City, IA, United States; ^5^School of Medicine, University of North Carolina, Chapel Hill, NC, United States; ^6^Department of Mechanical and Aerospace Engineering, College of Engineering, North Carolina State University, Raleigh, NC, United States; ^7^Department of Clinical Sciences, College of Veterinary Medicine, North Carolina State University, Raleigh, NC, United States; ^8^Department of Biological Sciences, North Carolina State University, Raleigh, NC, United States; ^9^Department of Population Health and Pathobiology, College of Veterinary Medicine, North Carolina State University, Raleigh, NC, United States; ^10^Department of Biological Sciences and Pathobiology, Immunology, University of Veterinary Medicine Vienna, Vienna, Austria

**Keywords:** eosinophils, histochemical analysis, porcine (pig) model, eosinophilic esophagitis, mast cell (MC), allergy

## Abstract

**Introduction:**

Accurate identification of eosinophils in tissue sections is required for diagnosis of eosinophilic esophagitis in humans and the assessment of severity of disease in allergy models. The pig may be a good model for sensitization and allergy models due to anatomical, physiological, and immunological similarities to humans. However, comparative studies on histochemical detection of eosinophils in fixed porcine tissue are lacking.

**Methods:**

Qualitative and quantitative comparisons were performed for six histochemical methods previously reported for eosinophil and mast cell detection in other species. Astra Blue/Vital New Red, Congo Red, Luna, Sirius Red, Toluidine Blue, and modified regressive Hematoxylin & Eosin were applied to formalin-fixed paraffin embedded full-thickness sections of porcine esophagus. Specimens were collected from young, crossbred pigs sensitized to ovalbumin with or without subsequent oral exposure to ovalbumin to produce eosinophilic esophagitis lesions for comparison to non-allergic controls.

**Results:**

Ease of eosinophil quantitation was analyzed, and varied by histochemical stain, to determine whether stain selection increased accuracy and efficiency of evaluation. Noticeable differences in color contrast between intracytoplasmic granules, surrounding tissue, and cellular components aided detection and identification of eosinophils and mast cells with Astra Blue/New Vital Red and Toluidine Blue, respectively. For eosinophils, Congo Red and H&E were adequate, while Luna and Sirius Red presented challenges for quantitation.

**Discussion:**

In this case, rapid and reliable characterization of porcine esophageal allergy models was made possible by using Astra Blue/New Vital Red for eosinophils and Toluidine Blue for mast cells.

## Introduction

Eosinophilic esophagitis (EoE), like many other food allergies and sensitivities, has become increasingly prevalent and significantly decreases quality of life. Between 10 and 19% of US adults have at least one diagnosable food allergy or suspected food allergy (1, 2) and the current prevalence of EoE has been estimated to be between 0.05 to 0.1% (3–11). EoE has been increasing in prevalence in parallel with food allergies and other diseases associated with allergic responses to foodstuffs. Foodstuffs most frequently implicated in food allergies include milk (12, 13), peanuts (14, 15), soy (16), wheat (17), egg, tree nuts, shellfish, and fish (18, 19) according to the FDA and literature reviews. Unfortunately, the diagnostic measure most often used for food allergies, IgE quantification (20), is insufficient to detect and/or monitor EoE because the association between inciting allergen and disease is not always clear. Due to the lack of reliable biomarkers, the assessment of EoE disease progression and characteristics relies upon histological analyses both clinically and in research animal models (21).

Although the precise mechanisms underpinning the disease are not clearly defined, eosinophils are primary pathogenic effector cells in EoE and the most used biomarker for diagnosis and monitoring of treatment response. Following an inflammatory response to allergen in the esophagus, mediators such as eotaxin-3 (22) are released, which bind primarily to the chemokine receptor CCR3 (23, 24) on eosinophils leading to their recruitment from circulation into the esophageal epithelium. Eosinophils accumulate in the subepithelial stroma as individualized cells or in clusters which may form eosinophilic pustules or eosinophilic layering (9, 25). Chronic eosinophilia leads to increased deposition of collagen, fibrosis, and stricture formation (26) contributing to clinical symptoms of dysphagia and food impaction in adults. Chronic cases of EoE are typified by regular relapses, persistent inflammation, and fibrostenotic sequelae (25) that require esophageal endoscopy and biopsy collection for subsequent histological diagnosis and disease monitoring. Similarly, mechanistic studies using animal models rely on histology endpoints and many models require cell enumeration in histology samples as a primary measure of disease progression and therapeutic efficacy. Histologic evaluation of human and animal tissues uses modified regressive hematoxylin and eosin (H&E) staining and standardized methods for identification of allergy effector cells, primarily eosinophils and mast cells. However, this approach to characterize the inflammatory response is time-consuming and requires significant training to avoid error.

**Figure 1:**
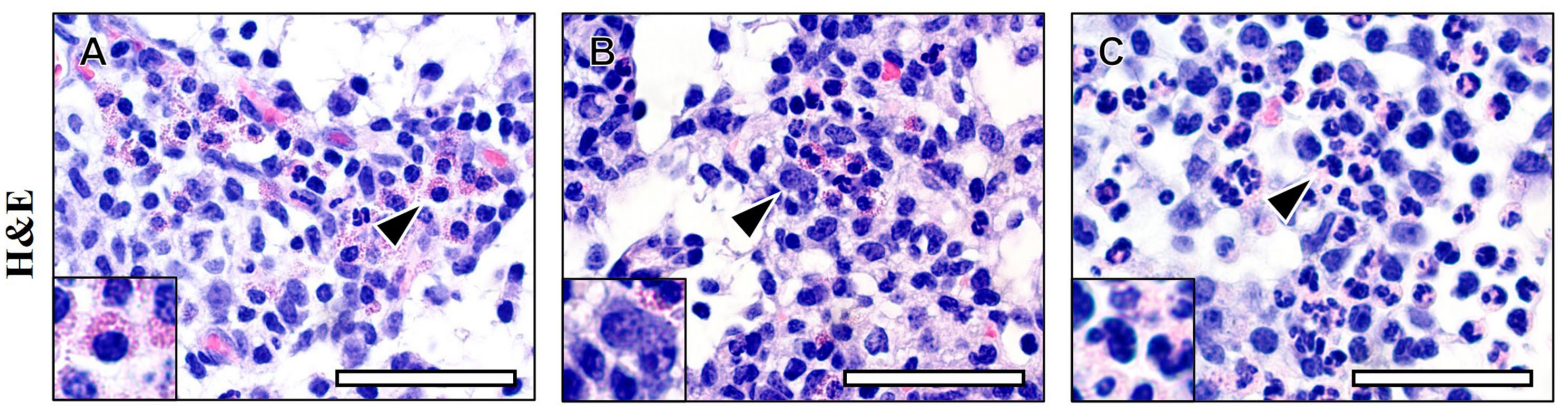
Eosinophil and mast cell detection by standard histochemical staining. Modified regressive hematoxylin and eosin (H&E) staining was applied to reference standards of porcine lymph nodes containing eosinophils **(A)**, mast cells **(B)**, and neutrophils **(C)**. Eosinophil and mast cell enumeration is laborious and may be compromised by lack of visual detection due to limited color contrast between cell types and/or surrounding tissues. Arrowheads = representative cell of interest. Scale bar = 100 microns.

To a highly trained observer, porcine eosinophils and mast cells can be identified when stained with H&E in tissue sections and, generally, discerned from other inflammatory cells (Figure 1). Comparable to mast cells from rodents and other mammals, porcine mast cells stained with H&E contain multiple distinct <2 μm intracytoplasmic basophilic granules. Additionally, porcine mast cells have a round central or paracentral nucleus (27). However, while H&E stains provide a versatile approach to assess tissues, the features of mast cells highlighted by histochemical staining is affected by fixation and staining technique (28, 29).

Similarly, detection of porcine eosinophils in H&E sections is laborious and, at times, challenging depending upon the visual contrast between eosinophils and supporting stroma or surrounding cell types. Surprisingly, detection of porcine eosinophils is more challenging compared to many other species because they lack the distinctive bilobed nuclei, which aids in eosinophil detection in many species. The rounded circular nuclei in porcine eosinophils resemble monocyte nuclear morphology and thus can be difficult to distinguish from either neutrophils or debris-laden monocytes (particularly dendritic cells [histiocytes]). Porcine eosinophils contain eosinophilic granular cytoplasm like rodent eosinophils (30). However, porcine eosinophils contain a central to eccentric moderate-sized nucleus that is most frequently rounded and only occasionally bilobed (31). Eosinophils and neutrophils in many species have some overlapping morphologic characteristics (30, 32, 33) as well. So morphologic differentiation between eosinophils and other cell types has been an ongoing issue precluding rapid microscopy examination, interpretation, and reporting across species. While H&E can be used to detect eosinophils in tissue (34), various histochemical methods have been employed to aid the identification and quantification of these leukocytes in tissue sections of various species (30, 35). However, we are not aware of the precedence for histochemical staining to optimize detection of eosinophils in porcine tissue sections.

To better study EoE disease, there is a critical need for development of reliable animal models of the disease and, ideally, standardized histological staining and procedures that improve detection and characterization of the inflammatory environment. Toward standardization of comparative animal models for disease, animal models should use metrics comparable to those for diagnosis of human EoE (9, 36, 37). Our group has developed porcine models of EoE-like disease referred to as Oesophageal / Esophageal Eosinophilia (EE) (38) and EoE (39), which could provide useful models for studying mechanisms of disease and translational approaches for treatments. As a follow-up study, we have utilized available tissue from subsets of the pigs that were selected because they spanned the full-range of eosinophilic inflammation to compare histological methods best suited for porcine eosinophil and mast cell assessment during eosinophilic inflammation in a pig model of EoE (39). Tissues were taken from controls and the highest responders in the EoE group to enable comparisons of histological techniques.

## Materials and methods

### Allergic sensitization and challenge

We utilized tissues generated from the development of eosinophilic esophagitis-relevant models in pigs that have been previously described (38, 39). For this study, we assessed available tissues from controls and selected a subgroup of the highest responders from the ovalbumin sensitized and challenged group that had marked eosinophilic inflammation. All animal use was approved by the Institutional Animal Care and Use Committee at North Carolina State University (19-729-B).

### Tissue sample preparation

Esophagus tissues and control lymph nodes were incubated in neutral-buffered formalin (NBF) for 24–72 h for fixation followed by tissue trimming and paraffin-embedding. Serial sections cut 5 μm thick with a microtome were transferred onto Superfrost Plus glass microscopy slides (Thermo Fisher Scientific, Waltham, MA United States) and air-dried at 37°C incubator. Slides were baked at 58°C for 45 min. The tissues were deparaffinized through xylenes, rehydrated in graded ethanol, and rinsed in distilled water before the staining protocols were performed. Tissues on slides were dehydrated in graded alcohols, cleared in xylene, and coverslip was applied with Permount (VWR, Radnor, PA, United States) mountant media. All histochemical methods were followed according to their original citations unless specifically stated and we were able to optimize the method in preliminary studies.

### Histochemical staining protocols

Tissues on microscope slides were stained with the previously described staining protocol for H&E (30), Astra Blue/Vital New Red (ABVR) (40), Congo Red (41), modified Sirius Red (30), Luna’s modification to the iron hematoxylin-biebrich scarlet protocol (42), or Toluidine Blue (42, 43). Specific details on each stain are included in the methods section of the Supplementary material.

### Qualitative assessment of eosinophil and mast cell staining

Qualitative assessments of staining parameters were performed by three pathologists. Eosinophils, mast cells, and other resident or infiltrating cells of hematopoietic origin (primarily myeloid cells) were examined qualitatively by H&E and special stains. In addition to the cells of interest, multiple background tissues and structures within tissues were examined for adequate qualitative levels of contrast which, in turn, impacts the ease of target cell detection and identification. Therefore, cells and tissue architecture from superficial esophageal epithelium to deep supporting stroma were examined. Control tissues from a diagnostic case submission of lymphadenopathy with high numbers of eosinophils were stained to provide accurate references for characteristics of lymphocytes, eosinophils, neutrophils, mast cells, and tissue architecture.

### Eosinophil quantification

Eosinophils were quantified in accordance with standards of diagnosis defined for clinical EoE cases in humans (44) adopted and described in previous porcine EoE model (38, 39) studies with similar histochemical techniques reported in other species (30). Histologic analysis was performed by a veterinary pathologist who was blinded to experimental groups (45). Microscopic examination, images from slides, and measurements were collected with image analysis software (AmScope v4.8) operating a high-resolution 14MP MU1400B digital camera (AmScope, ToupTek Photonics, CN) imaging system-equipped BX41 light microscope (Olympus, JPN). Before imaging, the system was calibrated with the use of a stage micrometer. Inflammatory cell recruitment was scored from 0 through 5 as follows: 0 = few scattered resident inflammatory cells; baseline; 1 = recruitment of scattered inflammatory cells within lamina propria and perivascular locations; 2 = few clusters of inflammatory cells in lamina propria and perivascular locations (mild); 3 = multiple clusters and coalescing clusters of recruited inflammatory cells in lamina propria and perivascular locations (moderate); 4 = clusters of recruited inflammatory cells in lamina propria with invasion of scattered cells into the overlying epithelium; and 5 = clusters of recruited inflammatory cells in lamina propria with intraepithelial microabscess formation or eosinophil layering (Table 1). Proportion of eosinophils per total inflammatory infiltrates were enumerated per histologic section of esophagus. Eosinophil counts were tallied per HPF at 200x magnification (for a high-power field area of 0.24 mm^2^) because those metrics were used for an EoE model in pigs (38, 39) and this is the most common microscope field size reported in the human EoE literature (44). Similarly, mast cells identifiable by prominent granules were enumerated per HPF at 200x magnification with Toluidine blue stain (i.e., for a high-power field area of 0.24 mm^2^).

**Table 1:** Inflammation severity scoring for esophageal eosinophilia.

Inflammation score	Severity	Description
0	None	No detected eosinophils
1	Minimal	Few scattered interstitial eosinophils
2	Mild	Clusters of <15 interstitial eosinophils per 0.24mm^2^
3	Moderate	Clusters of 15–30 interstitial eosinophils per 0.24mm^2^
4	Marked	Intraepithelial eosinophils or clusters of >30 interstitial eosinophils per 0.24mm^2^
5	Severe	Layering of intraepithelial eosinophils or clusters of >45 interstitial eosinophils per 0.24mm^2^

### Graphics and statistical analysis

GraphPad Prism (GraphPad Software, La Jolla, CA, United States) software was used for statistical analysis and produced graphics for figures. Assessment of eosinophil detection for each stain was performed with a linear mixed-model analysis for repeated measures. This model was chosen for its ability to detect differences between each of the selected staining methods. The mixed-model analysis was used, where possible, to account for the correlation from within the same tissue specimens as well as between groups, including the potential effect of staining method on eosinophil enumeration and inflammation scoring. To assess statistical significance the following tests were used: One-way ANOVA with Sidak post-test for multiple comparisons, linear mixed-model two-way ANOVA with Sidak post-test for multiple comparisons, Kruskal-Wallis two-way ANOVA with Mann–Whitney U-test comparison for significance, and *T*-test. Statistical significance was defined as *p* < 0.05 wherein * represents p < 0.05, ** represents *p* < 0.01, *** represents *p* < 0.001, and **** represents *p* < 0.0001.

**Figure 2:**
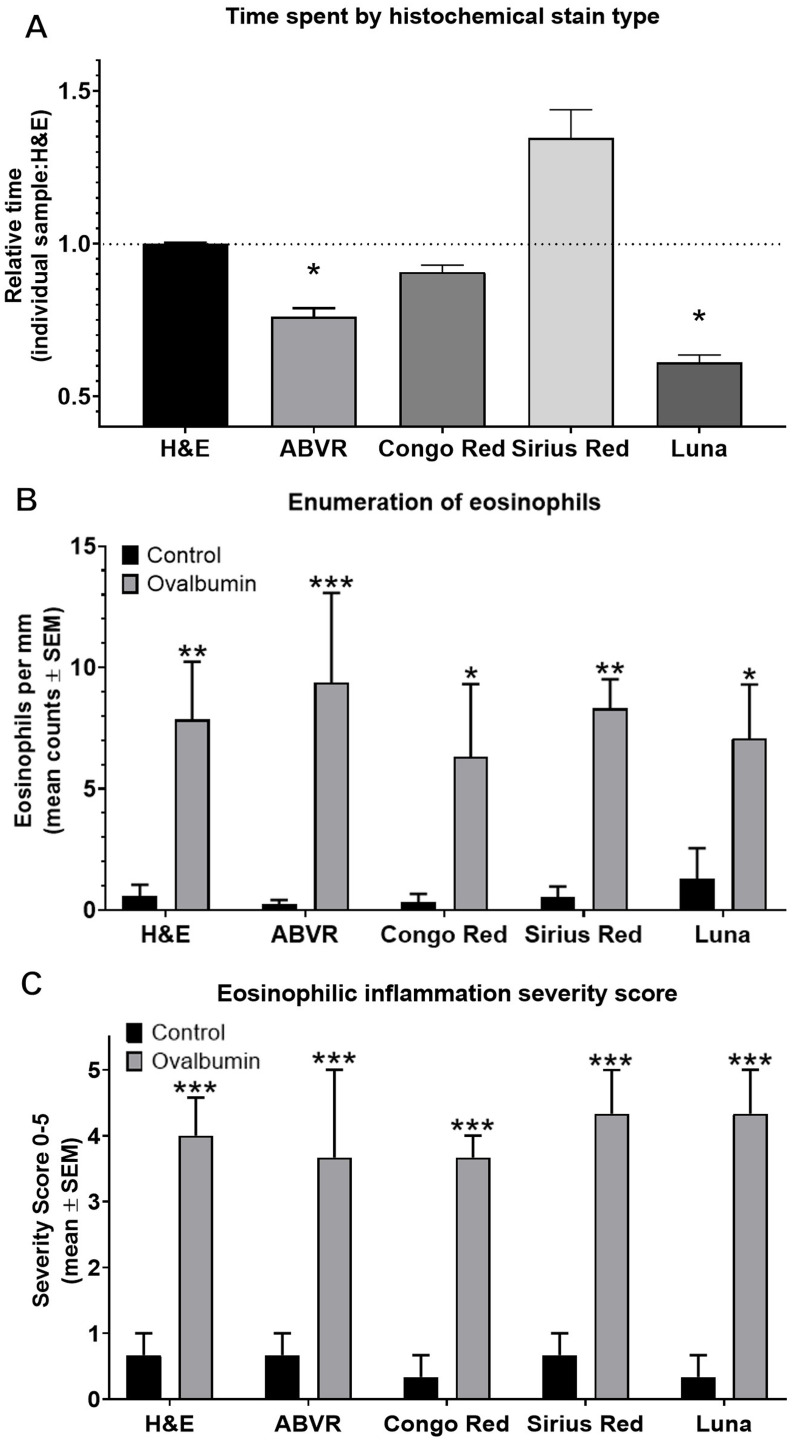
Detecting eosinophils in esophageal mucosa. Detection of mucosal eosinophils located in the epithelium or lamina propria was enabled by routine and special stains including H&E, ABVR, Congo Red, Sirius Red, or Luna stain. **(A)** Relative amount of time required for eosinophil enumeration adjusted to corrected for tissue size was significantly lower for ABVR (*p* = 0.0445) and Luna (*p* = 0.0159) stains compared to H&E which required 59.5 s/mm on average. **(B)** There was no significant difference in eosinophil counts by stain. Eosinophil counts were significantly higher in EoE pigs compared to controls by all stains including H&E (*p* = 0.0043), ABVR (*p* = 0.001), Congo Red (*p* = 0.0137), Sirius Red (*p* = 0.0028), and Luna (*p* = 0.0165). **(C)** Eosinophilic inflammation severity score was significantly higher in EoE pigs compared to controls by H&E (*p* = 0.0004), ABVR (*p* = 0.0009), Congo Red (p = 0.0004), Sirius Red (*p* = 0.0002), and Luna (*p* = 0.0001) stains.

## Results

### Eosinophil and mast cell detection by standard histochemical staining—H&E

Routine histochemical staining procedures for formalin-fixed paraffin-embedded tissues are nearly exclusively H&E. We demonstrate H&E staining of cells within porcine lymph node (Figure 1) as a representation of the challenges encountered performing histochemical staining. More specifically, the staining-associated visual similarities between either cell type and/or supporting tissue architecture hamper cell detection amongst surrounded tissues lacking differential staining (i.e., sensitivity) and distinguishing the cell of interest from other cells based upon staining characteristics of each cell type (i.e., specificity).

### Comparison of special stains for eosinophil quantitation in inflamed and non-inflamed esophageal mucosa

Histochemical stain performance was best evaluated in a model with variable numbers of eosinophils recruited into tissues to demonstrate the suitability of the stain. Here, we chose to highlight the use of special stains selected to enumerate low to high numbers of infiltrating eosinophils with tissue from high responder allergic model pigs compared to non-allergic controls. To evaluate our hypothesis that high contrast staining techniques for eosinophils can improve detection and reduce time required, microscopy slides were stained with H&E, ABVR, Congo Red, Sirius Red, and Luna stains. Notably, Toluidine Blue stain has not been included for eosinophil detection due to lack of utility; therefore, comparison was not made between H&E and Toluidine Blue for eosinophils. Time required for eosinophil enumeration using H&E required 59.5 s/mm on average (Relative time = 1). Corrected for tissue size, the relative time for ABVR (*p* = 0.0445) and Luna (*p* = 0.0159) stains were significantly lower (Figure 2A). Luna stain was the most effective at reducing time for analysis of sections.

Importantly, stain selection had no significant effect on slide interpretation and analysis of tissue eosinophilia (Figure 2B) and inflammation severity (Figure 2C). There was no significant difference in eosinophil counts per mm by stain (Figure 2B) nor inflammation score by stain (Figure 2C) when comparing only control specimens to control specimens of a different stain or only EoE specimens to an EoE specimen with a different stain. Nonetheless, the effect of stain selection on eosinophil detection in pig esophagus demonstrated a decreased time investment with ABVR (nearly 30%-time savings) and Luna (nearly 50%-time savings) stains compared to H&E. Collectively, data presented demonstrate that special stain selection can improve the rapidity of eosinophil detection thereby reducing time required for evaluation, without significantly reducing accuracy.

### Qualitative assessment of porcine eosinophils and mast cells with special stains

**Figure 3:**
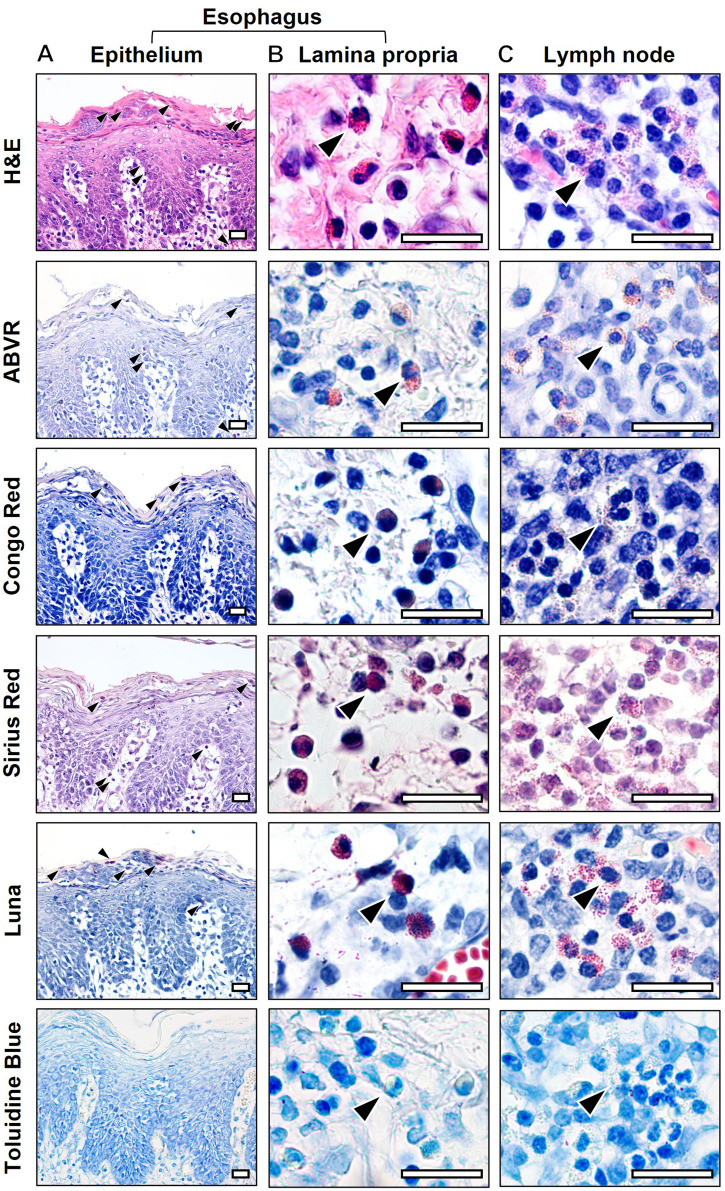
Eosinophil detection improved by histochemical special stain selection. Eosinophils (arrowheads) within the esophageal epithelium **(A)** and lamina propria **(B)** from the porcine esophageal eosinophilia model in addition to eosinophils from reference control porcine lymph node **(C)** were stained with either H&E, Astra Blue – New Vital Red (ABVR), Congo Red, Sirius Red, Luna, or Toluidine Blue stains. Scale bar = 50 μm.

The increased speed of analysis for histological samples is explained by the ease of differentiation between cell types and detection amongst the surrounding tissue. The assessment of specificity and accuracy of staining protocols for eosinophilic inflammation revealed that while the features of eosinophils were generally discernable with the standard H&E stain, it gave minimal contrast (Figure 3). Special stains, especially ABVR and Luna stains, improved contrast between eosinophil granules, neutrophil granules, and surrounding structures and improved not only the speed of detection (as shown in Figure 2) but also the reliability of results.

#### H&E stain

**Figure 4:**
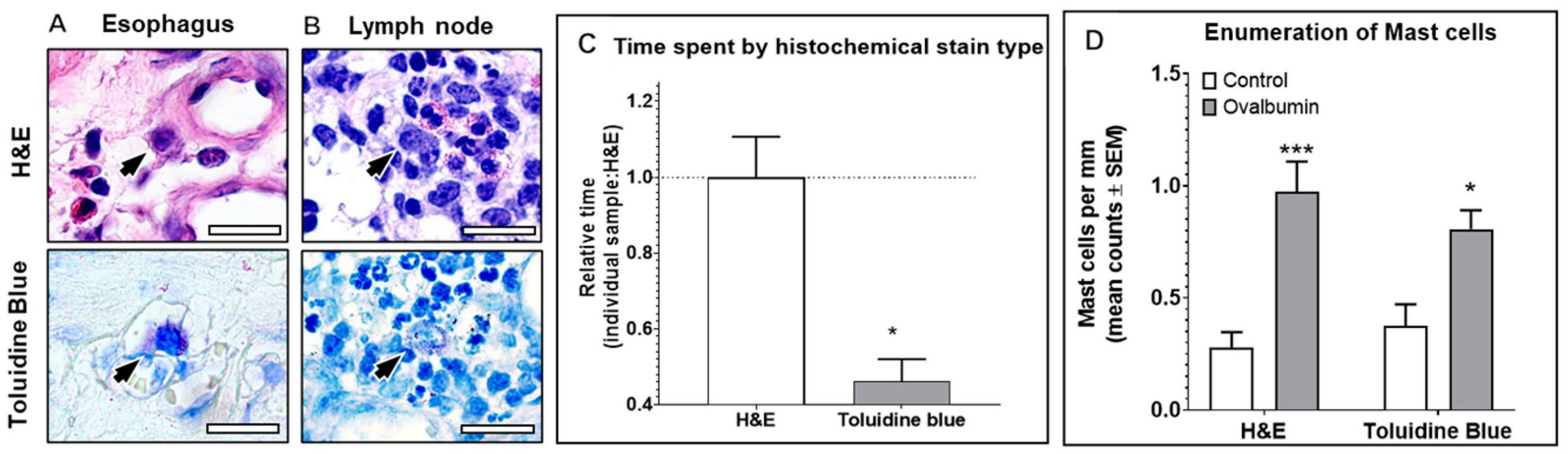
Mast cell detection improved by histochemical special stain selection. Mast cells (arrows) in esophageal lamina propria **(A)** from esophageal eosinophilia model and reference control porcine lymph node **(B)** were stained with either H&E or Toluidine Blue. **(C)** Relative amount of time required for mast cell enumeration adjusted to corrected for tissue size was significantly lower for Toluidine Blue stain (*p* = 0.0114) compared to H&E which required 79.2 s/mm on average. **(D)** Mast cell counts were not significantly different by either H&E or Toluidine Blue stains. Mast cell counts were significantly lower in EoE pigs compared to controls by H&E (*p* = 0.0006) and Toluidine Blue (*p* = 0.0286). Scale bar = 25 μm.

H&E stain provided adequate visual detection of key histologic features sufficient to distinguish between background components of tissues and cells of interest including subcellular components such as intracytoplasmic granules and cytoplasm. For eosinophils, intracytoplasmic granules were stained densely eosinophilic (i.e., dark reddish-pink) to practically orange-red (Figure 3), which somewhat aided detection. The eosinophilic granules contrasted somewhat with less densely stained surrounding stroma and cells. However, visual detection at lower magnification was challenging. For neutrophils, intracytoplasmic granules were pale eosinophilic and generally blended imperceptibly with the pale eosinophilic cytoplasm. The difference in dye uptake by granules of eosinophils and neutrophils was subtle. Similarly, mast cells were identifiable with H&E stain but not easily detectable without high power magnification in many cases (Figure 4). The similarity of color to many other cellular components (e.g., basophilic nuclei in every cell and uniformly basophilic cytoplasm of some cell types) can present a challenge and markedly slowed analysis of mast cells with H&E.

#### ABVR stain

Compared to H&E staining, ABVR stain provided increased visual detection with significant contrast between key histologic features sufficient to easily distinguish between background components of tissues and cells of interest including subcellular components of intracytoplasmic granules and cytoplasm. For eosinophils, intracytoplasmic granules stained brilliant to rose red (Figure 3), which aids in both detection and easy identification rapidly and efficiently. The red staining of eosinophil granules contrasted against royal blue of nuclei, the somewhat light blue to pink cytoplasm, and light blue to bluish-pink surrounding tissue architecture. More specifically, the visual identification of eosinophils was simple using low to moderate magnification with minimal confusion between eosinophils and other myeloid-lineage cells. For neutrophils, intracytoplasmic granules were vague pale pink and generally blended imperceptibly with the pale pink (very light red hues) of the cytoplasm. Lack of neutrophil granule distinction was an issue observed in H&E. The marked difference in dye uptake by granules of eosinophils and neutrophils with ABVR, evident even at lower magnification, contributed to more rapid microscopic evaluation compared to H&E. Similarly, mast cells were distinguished from eosinophils and neutrophils, but not because of stain uptake. Instead, mast cell granules were refractile with variable stain uptake ranging from non-staining to deep violet, which varied even within some granules and was not easily detectible without high power magnification. Therefore, although mast cells were distinguishable, identification was slower due to the need to switch to high power.

Regarding tissue architecture and coloration, ABVR stained nuclei royal blue, with somewhat light blue to pink cytoplasm, and light blue to bluish-pink for stroma in sections containing submucosal glands, nerve, artery, epithelium, and subepithelial stroma (Supplementary Figure 1).

#### Congo Red stain

Congo Red provided moderate contrast between eosinophil granules and surrounding structures. For eosinophils, intracytoplasmic granules stained red (Figure 3) and generally blended imperceptibly with the surrounding cytoplasm. For neutrophils, intracytoplasmic granules stained pale pink to red (Figure 3) and also blended imperceptibly with the surrounding cytoplasm. For mast cells, intracytoplasmic granules were indistinct and blue to violet (Figure 3). While color contrast was moderate between eosinophil granules and surrounding tissues, thereby improving ease of apparent detection, confounding factors preclude utility of the stain for this application. Eosinophil granules were markedly less prominent precluding distinction from each other in esophagus samples and only moderately differentiated from surrounding structures in lymph node samples. To increase contrast between intracytoplasmic granules and cytoplasm, the light intensity had to be increased during microscopic examination to the point of discomfort in viewing. Such increased light intensity increases blue light-wavelength-related eye strain. Whilst somewhat visible to human eyes, the smudgy reddish granules had slight refractile edge in esophagus samples (Figure 3) that was not easily detected by early attempts with computer vision algorithms either.

Other cells stained similarly to eosinophils when using Congo Red. Intracytoplasmic staining of neutrophil granules had a similar smudgy reddish hue when compared with eosinophil granules. Given the similar color intensity (i.e., pallorous) and indistinct margins, these features provided eosinophils and neutrophils with very similar morphologic appearance despite known differences visible even by H&E. Visible similarities precluded rapid, accurate enumeration. Additionally, mast cell granules were generally refractile and non-staining to pale pink, appearing so faint that it was challenging to distinguish from the royal blue to violet cytoplasm.

Congo Red provided tissue architecture with light blue to bluish-pink for stroma color in sections containing submucosal glands, nerve, artery, epithelium, and subepithelial stroma (Supplementary Figure 1). By contrast, Congo Red stained nuclei royal blue, with somewhat light blue to pink cytoplasm lending the entire slide to appearing vaguely uniform colors throughout failing to draw the eye to salient features.

#### Sirius Red stain

Sirius Red obscured salient histologic features compared to other stains. All background tissue ultrastructure was vague pink-brown to pale violet-brown of varying darkness. Similarly, individual cells and cellular features lacked contrast as the entire tissue and cells stained with various hues of the same vague color palette of pink-brown. Eosinophil enumeration was complicated by the lack of differential staining between eosinophils, neutrophils, and mast cells. Granules of eosinophils were pale red and somewhat distinct, and the granules of neutrophils were indistinguishable from eosinophil granules by color. Therefore, neutrophils and eosinophils appeared similar at lower magnification. Whilst 5 μm tissue sections typically present with crisp enough edges to discern subcellular features and tissue architecture, higher power magnification (e.g., 200x and 400x) was frequently used to detect and positively identify cell types. Repeated magnification changes limit visual fields to smaller areas making the task of microscopy laborious with Sirius Red.

Similarly, mast cell granules were minimally refractile and pale to dark pink-brown to vaguely violet (i.e., a combination of hues resembling both background and the nucleus of all cells). Although mast cells are typically larger than eosinophils, the variable morphology of porcine eosinophil’s nucleus, which frequently lacks lobulation, complicated distinction between the two cell types. Distinguishing between eosinophils, neutrophils, and even mast cells with Sirius Red stain was extremely laborious.

Regarding tissue architecture and coloration, the Sirius Red technique stained nuclei pale to dark pink-brown to violet-brown, with pink-brown cytoplasm, and light pink-brown for stroma in sections containing submucosal glands, nerve, artery, epithelium, and subepithelial stroma (Supplementary Figure 1).

#### Luna stain

Luna stain complicates determination of eosinophil enumeration because mast cells are typically known to have Luna-positive staining granules with some versions of Luna stain. In our hands, the Luna stain protocol we used (see Supplementary material) provided increased visual detection of eosinophils with significant contrast between key histologic features sufficient to easily distinguish between background components of tissues and cells of interest including subcellular components of intracytoplasmic granules and cytoplasm. For eosinophils, intracytoplasmic granules stained brilliant to crimson red (Figure 3), which aided both rapid detection and easy identification of eosinophils. The red granules contrasted against the blue nuclei, light blue cytoplasm, and light blue of surrounding tissue architecture. More specifically, the visual identification of eosinophils was simple by low to moderate magnification. However, further time-consuming evaluation was necessary on each slide to differentiate between positive-staining cell types.

For neutrophils, intracytoplasmic granules were extremely pale to pale pink and generally blended imperceptibly with the pale pink of the cytoplasm, comparable to appearance with H&E. The marked difference in dye uptake by granules of eosinophils and neutrophils provided increased contrast between these cell types even at lower magnification, markedly increasing the speed of microscopic evaluation. However, mast cells were easily confused with eosinophils. Mast cells can be differentiated from eosinophils with Luna stain due to subtle differences in dark crimson red to dark reddish-violet granules, but positive identification was challenging in the pig. Porcine eosinophils and mast cells had similar features with Luna stain including a single non-lobulated nucleus and prominent dark red granules. Subtle size differences can assist identification, but assessing size is laborious and inherently inaccurate when neighboring cells also contain granules. Simply, one cannot easily distinguish between mast cells and eosinophils consistently in the pig with Luna stain applied herein.

#### Toluidine Blue stain

Toluidine Blue is a common stain used to identify mast cells in tissue in many species. As expected, Toluidine Blue provided increased contrast between mast cell granules and surrounding structures in porcine tissue architecture. Eosinophil and neutrophil granules were generally non-staining to pale pink, so faint that it was challenging to distinguish from pale blue cytoplasm (Figure 3). Mast cells were easily detected in the esophagus (Figure 4A) and lymph nodes (Figure 4B). Mast cells were distinguished from eosinophils with Toluidine Blue with mast cells exhibiting distinct, highly contrasting “metachromatic” granules. Mast cells were easily distinguished from neutrophils as neutrophils had nonspecific vague intracytoplasmic staining and multilobulated nuclei. Mast cells were distinguished from histiocytes as histiocytes lacked prominent granules and generally lacked intracytoplasmic accumulation of eosinophilic material except for a few debris-laden histiocytes. One caveat, however, from the authors’ experience is that some positive-charged exogenous substances can lead to well-circumscribed, intracytoplasmic [intravesicular] accumulation within debris-laden histiocytes. Enumeration of mast cells using Toluidine Blue stain was much easier with more rapid analysis of tissues (Figure 4C). Other than the significant increase in speed, staining with Toluidine Blue did not significantly affect the interpretation of slides or numeration of mast cells compared to H&E (Figure 4D). Conclusively, Toluidine Blue facilitated the enumeration of mast cells but was not adequate for neutrophil or eosinophil quantification.

## Discussion

This study provides qualitative and quantitative comparisons for six histochemical methods on porcine tissues. Histochemical stains were selected based upon prior use in other species for detection of key cells in allergic inflammation - eosinophils and mast cells. We investigated the use of H&E, ABVR, Congo Red, Luna, Sirius Red, and Toluidine Blue in swine tissue selected from a model of EoE to span the eosinophilic inflammation range. ABVR and Luna stains provided increased speed of detection and ease of enumeration for eosinophils. Congo Red provided moderate contrast. Sirius Red and H&E provided minimal contrast between eosinophil granules, neutrophil granules, and surrounding structures. While ease of eosinophil quantitation varied significantly in porcine tissues, in our assessment, we established that the use of ABVR for eosinophils and Toluidine Blue for mast cells most improved both positive detection and rapid enumeration of specific cells yielding both reliable and reproducible results.

Detection and/or quantification of eosinophils in tissues are important for diagnostics and for researchers investigating mechanisms of disease and assessment of novel therapeutics in allergic disease models, such as EoE. However, observing qualitative and quantitative differences in staining applications in porcine tissues has been lacking. This study confirms preferred techniques in other species for the assessment of tissues in swine models of allergy, which is timely considering the increased use of pigs in biomedical research. In the EoE model that we used for tissues, all candidate histological methods were useful to detect increased eosinophils and mast cells as a proxy for eosinophil and mast cell recruitment. Interestingly, there was slight variation in the absolute enumeration of eosinophils between staining methods despite serial sectioning through the same tissues. There are several possible explanations that could account for such variations in the specificity and sensitivity of each technique. First, each stain contains dyes with affinity for different subcellular components (Table 2 and Supplementary material) within granules of eosinophils and mast cells. Chemical properties of dyes likely cause variations in uptake and, therefore, affect detection (30). Second, the presence of nonspecific staining of myeloid-lineage cells could, in some instances, contribute to elevated eosinophil enumeration. This circumstance has been proposed for neutrophils appearing like eosinophils (30) with Sirius Red and Congo Red. Similarly, we observed eosinophils appearing like mast cells in the Luna stain, this made distinguishing the two cell types difficult. Mast cells can be differentiated from eosinophils with Luna stain due to subtle differences in dark crimson red to dark reddish-violet granules, but positive identification was challenging. This is an issue with porcine tissues where nuclear morphology of eosinophils resembles that of mast cells, where in other species the nuclear morphology would help differentiate the cell types.

**Table 2:** Color characteristics of eosinophils, neutrophils, and mast cells per stain method.

Histochemical stain method	Abbreviation	Eosinophil’s intracytoplasmic granules	Neutrophil’s intracytoplasmic granules	Mast cell’s intracytoplasmic granules	Dye characteristics
Hemotoxylin and eosin	H&E	Distinct moderately eosinophilic (pink) granules	Indistinct eosinophilic (pink) cytoplasmic staining	Dark violet granules	Basic and acidic dyes, bind lysine and arginine-rich proteins (52)
Astral Blue with Astral Vital New Red	ABVR	Distinct brilliant to rose red granules	Indistinct faint to pale pink cytoplasmic staining	Non-staining to deep violet, refractile granules	Metallo-phthalocyanine cationic chromophore (51, 52)
Congo Red	Congo	Indistinct red granules	Indistinct pale pink to red cytoplasmic staining	Indistinct blue to violet granules	Azo (dis-azo) cationic chromophore (52); Hydrogen bonding of azo-amine groups to hydroxyl radicals of eosinophils (53)
Sirius Red	Sirius	Distinct to indistinct to pink-brown to red-brown granules	Indistinct pink-brown cytoplasmic staining	Distinct to indistinct Violet-brown granules	Cationic chromophore; Azo (polyazo) dye (52)
Luna’s stain	Luna	Distinct brilliant to crimson red granules	Indistinct pale pink granules	Violet to red granules	Cationic chromophore (51)
Toluidine Blue	TBlue	Distinct non-staining, refractile granules	Indistinct, nonstaining granules	Dark violet, metachromatic granules	Cationic chromophore; Thiazine dye (52)

As mentioned in the qualitative assessment, nonspecific background tissue and variable staining pattern (e.g., cell-to-cell variation) drastically diminishes qualitative features. On the other hand, increased contrast between background and cells or distinct cell features (e.g., distinct granules in eosinophils contrasted with diffuse homogenous staining in neutrophils) and morphology are useful to prevent this detection artifact. To counteract issues encountered with progressive H&E histochemical stain, enhanced staining of eosinophils was addressed through our H&E protocol collection, referred to as “modified regressive H&E” in this case (see Supplementary material). Enhanced staining improves detection as the intensity of various hues within the granules is intensified thereby increasing contrast. For H&E, the pink and violet hues of stain binding to the granules provides a slightly more intense pink to reddish hue giving visual contrast to granules. While the H&E protocol was selected as the best fit-for-purpose stain, other histochemical stains outperformed our modified regressive H&E.

ABVR was ultimately superior for eosinophils due to the increased contrast of red granules compared to nearly every other cellular component and tissue component that lacked that intensity of red hue (e.g., blue hues and vague violet) as previously described in other species (30, 40). ABVR was the best stain for eosinophils and mast cells could also be identified. However, variation in mast cell appearance with ABVR in porcine tissue, at least in our hands, slowed down the identification of mast cells. Therefore for rapid identification of mast cells, in agreement with a prior porcine study (27), Toluidine Blue provided increased contrasting hues most appropriate for mast cell staining even in partially degranulated mast cells. Ultimately, user-friendliness of any stain technique depends on the application of simple chemical principles regarding dye affinity and physical principles of those dyes on the tissue resulting in a spectrum of color hues, intensity, and contrast that aids detection and unequivocal identification (sensitivity and specificity).

The primary objective of this study was to establish histological methods that could not only characterize porcine eosinophilic inflammation but also provide the most efficient and reliable method for quantitation of eosinophils and mast cells in porcine tissues. Luna stain provides the fastest method for eosinophil analysis. However, there is the caveat that some mast cells may be counted as eosinophils in porcine tissue when using Luna stain. Luna stain provides excellent contrasting stain characteristics between eosinophils and neutrophils, and background tissue lending to ease and rapidity of detection and identification generally at lower magnification. Luna also stains the most superficial layers of esophagus pale, to dark, crimson red which may increase the time required for microscopic examination at low magnification when scattered eosinophils infiltrate superficial epithelium. Therefore, the authors would recommend Luna stain for a rapid count of porcine eosinophils with the caveat that mast cells may also be inadvertently counted to demonstrate increased cell infiltration into tissues as a proxy of local allergy response.

Moving forward, it would be interesting to apply our observations to designing and testing artificial intelligence strategies to enumerate mast cells and eosinophils in histology sections using the special staining protocols we have employed and assessing their accuracy and utility. AI for histological diagnosis, assessment of severity or enumeration of eosinophils and mast cells in EoE is a fast-growing area of interest (46–49). However, AI has mostly relied upon more expensive and time-consuming staining methods with antibody-based EPX or tryptase staining protocols. We envisage that AI could be developed to utilize sections with the special stains we have employed provided careful training and validation were performed. Luna stain could be a relatively cheap and quick special stain candidate for AI-based enumeration of eosinophils in porcine tissue due to the high contrast staining even at low power. Furthermore, future studies comparing the special staining methods we have employed with IHC staining in porcine tissue are needed to assess the most consistent and reliable method for cell enumeration and AI analysis in porcine EoE models.

## Conclusion

We have determined optimal stains for eosinophil and mast cell enumeration in swine (e.g., esophagus and lymph node). For our purposes, ABVR and Toluidine Blue stains were identified as most useful with the caveat acknowledging utility and rapidity of the Luna stain in certain circumstances. Accurate identification of eosinophils in tissue sections, a representation of relative eosinophil recruitment, dictates diagnosis of eosinophilic esophagitis in humans and severity of disease in many allergy models. Here, we provide the most accurate histochemical stain for porcine eosinophils and porcine mast cells which also decreases the need to use expensive immunohistochemical methods. In our hands, the optimal histochemical stains for detection and enumeration of key allergic effector cells were as follows: ABVR for eosinophils, Toluidine Blue for mast cells, and Luna for rapid combined counts of mast cells and eosinophils. Improved detection and enumeration of the key indicators of allergic inflammation gives reliable and reproducible results in a swine model of EoE. The findings are timely given the increasing use of pigs in biomedical research (50) owed to anatomical, physiological, and immunological similarities with humans which, in turn, increases the need for comparative studies on histochemical stains.

## Data Availability

The original contributions presented in the study are included in the article/Supplementary material, further inquiries can be directed to the corresponding author.
