# Supplementary material for: Comparison of histochemical methods for the analysis of eosinophils and mast cells using a porcine model of eosinophilic esophagitis
Source: Front Vet Sci. 2025 Mar 19;12:1540995. doi: 10.3389/fvets.2025.1540995 (PMC11963769; doi:10.3389/fvets.2025.1540995)
Supplement: Supplementary file 1 [file Data_Sheet_1.docx]

**Supplement**

**Comparison of histochemical methods for the analysis of eosinophils and mast cells using a porcine model of eosinophilic esophagitis**

Douglas B. Snider^1,2,3^, David K. Meyerholz^4^, Evan S. Dellon^5^, Lizette M. Cortes^1,2^, Akash Karri^6^, Anthony T. Blikslager^2,7^, Scott Laster^2,8^, Tobias Käser^2,9,10^, and Glenn Cruse^1,2^*

**Affiliations**

^1^ Department of Molecular Biomedical Sciences, College of Veterinary Medicine, North Carolina State University. Raleigh, NC 27607, USA

^2^ Comparative Medicine Institute, North Carolina State University. Raleigh, NC 27607, USA

^3^ Comparative Medicine and Translational Research Training Program, North Carolina State University. Raleigh, NC 27607, USA

^4^ Department of Pathology, University of Iowa Carver College of Medicine, Iowa City, IA, USA

^5^ University of North Carolina, School of Medicine, Chapel Hill, NC, USA

^6^ Department of Mechanical and Aerospace Engineering, College of Engineering, North Carolina State University. Raleigh, NC 27607, USA

^7^ Department of Clinical Sciences, College of Veterinary Medicine, North Carolina State University. Raleigh, NC 27607, USA

^8^ Department of Biological Sciences, North Carolina State University. Raleigh, NC 27607, USA

^9^ Department of Population Health and Pathobiology, College of Veterinary Medicine, North Carolina State University. Raleigh, NC 27607, USA

^10^ Department of Biological Sciences and Pathobiology, Immunology, University of Veterinary Medicine Vienna. 1210 Vienna, Austria

*To whom correspondence should be addressed:

Glenn Cruse, PhD, Associate Professor, Department of Molecular Biomedical Sciences, College of Veterinary Medicine, North Carolina State University. Biomedical Partnership Center, 1060 William Moore Drive, Raleigh, NC 27607. Email: gpcruse@ncsu.edu. Phone: +1.919.515.8865.

**Supplementary Materials and Methods**

***Modified Regressive H&E staining protocol***

The modified regressive H&E (referred to as modified H&E) histochemical staining protocol^1^ was performed with modification of the eosin staining step. In the regressive H&E stain, selection of an acid-free Harris hematoxylin and bluing step provides finite control of darkness of basophilic tissues thereby improving clarity by increasing contrast between light and dark violet hues. Modifying the eosin staining was previously described for staining of rodent eosinophils^2^ and has the advantage of minimizing background tissue eosin staining thereby increasing contrast between light and dark pink hues and improving detection of eosinophils^3^. The unstained, deparaffinized, rehydrated tissues on microscope slides were immersed in pre-filtered, acid-free Harris hematoxylin 95057-858 (VWR, Radnor, PA, USA) for five minutes then gently rinsed in tap water. The Harris hematoxylin must be previously filtered through #4 Whatman filter, previously stored in complete darkness, and less than 6-9 months old. The slides were placed in 0.3% acid alcohol for three to five dips at 1 second intervals. Slides were gently rinsed in tap water for two minutes. Bluing was performed by immersing slides in pH 10, 0.2% ammonium hydroxide (v:v) in deionized water for 30 seconds to one minute and gently rinsed with tap water. Most importantly, quality checks are needed to determine length of time for bluing (i.e. 30 to 60 seconds) because hematoxylin will ripen from time (within 6-9 months) markedly changing the final color. Slides were immersed in pH 4.4 Eosin Y (Surgipath Medical Industries, Richmond, IL, USA) for 20 to 60 seconds. Finally, slides were rinsed for 2 minutes in tap water.

***Astra Blue/Vital New Red (ABVR) staining protocol***

The ABVR histochemical staining protocol^4^ was performed with minimal modification. In our studies, the unstained, deparaffinized, rehydrated tissues on microscope slides were immersed in Astra Blue (Millipore Sigma, Burlington, MA, USA) for thirty minutes at room temperature before being rinsed in running tap water. Subsequently, slides were immersed in Vital New Red (Pfaltz and Bauer, Waterbury, CT, USA) for thirty minutes and rinsed in running tap water. The sections were counterstained in Harris hematoxylin (VWR, Radnor, PA, USA) for five seconds. Slides were gently rinsed in tap water. Bluing was performed by immersing slides in pH 10, 0.2% ammonium hydroxide (v:v) in deionized water for 1 minute and gently rinsed with tap water.

***Congo Red staining protocol***

The Congo Red histochemical staining protocol^5^ was optimized to improve eosinophil detection through longer exposure to the stain, creating mild blue counterstain and darker red stain than the original protocol. The unstained, deparaffinized, rehydrated tissues on microscope slides were immersed for five minutes in Gill’s double strength hematoxylin (Polysciences, Warrington, PA, USA) followed by a gentle rinse in running tap water using a squirt bottle with tilting of slides at a 45° angle. Sections were then stained with prefiltered 0.5% Congo Red (w:v) (Millipore Sigma, Burlington, MA, USA) in deionized water for fifteen minutes. Slides were rinsed with deionized water to prevent precipitate formation.

***Sirius Red staining protocol***

The Sirius Red stain protocol^6^ was modified as previously described^2^ including elimination of the sodium chloride step. In our studies, the unstained, deparaffinized, rehydrated tissues on microscope slides were immersed in Harris hematoxylin (VWR, Radnor, PA, USA) for two minutes. Slides were rinsed in running tap water followed by a rinse in 100% ethanol. The sections were immersed in an alkaline (pH 8–9) 1% sirius red (w:v) (Millipore Sigma, Burlington, MA, USA) in 50% ethanol (v:v) solution in deionized water. Finally, slides were rinsed for 2 minutes in tap water.

***Luna’s Iron Hematoxylin-Biebrich Scarlet staining protocol***

In our studies, the Iron Hematoxylin-Biebrich Scarlet protocol reported by Luna^7^ for identification of eosinophils was used. To avoid confusion, recall that the term “Luna” referring to Luna’s protocol is vague and can refer to range of modifications including toluidine blue dye^7,8,9^ commonly used for detection of mast cells or other stains including Luna’s Aldehyde Fuschin and Luna’s Methylene Blue^8^ (Luna, 1992). In our studies, the unstained, deparaffinized, rehydrated tissues on microscope slides were immersed for five minutes in a working solution of Weigert’s iron hematoxylin-Biebrich Scarlet solution (Rowley Biochemical, Danvers, MA USA) composed of 1% hematoxylin (w:v) in 95% ethanol mixed with equal parts of pH 6.5 1% ferric chloride (w:v) in 95% ethanol added to 1% Biebrich Scarlet (w:v) in deionized water. Subsequently, the slides were immersed for one second intervals eight times (i.e. eight seconds) in 1% acid alcohol solution with deionized water for differentiation. Slides were gently rinsed in running tap water using a squirt bottle with tilting slides at a 45° angle for 2 minutes. Sections were then dipped for one second intervals five times (i.e. five seconds) into lithium carbonate solution until tissue sections began to turn blue. At that time, microscopy was performed to confirm eosinophil granules and erythrocytes appeared bright red while mast cell granules appeared violet to red. Slides were washed in running tap water for two minutes.

***Toluidine blue staining protocol***

In our studies, toluidine blue protocol reported previously^9^ was used for identification of mast cells. To avoid confusion, recall that the author “Luna” published many staining protocols^7,8^; however, we will herein refer to this protocol as Toluidine blue to avoid the fray. In our studies, the unstained, deparaffinized, rehydrated tissues on microscope slides were immersed for two to three minutes incubation in a working solution of Toluidine blue composed of 5mL of a 1% Toluidine blue O (w:v) in 70% ethanol mixed with 45mL of a pH 2.3 (adjusted with HCl) formulated to 1% sodium chloride solution (w:v) in deionized water. Typically, the test slide with fixed tissue is stained in toluidine blue solution for two and one-half minutes then evaluated at the end of the protocol when the decision is made to either decrease to two minutes, remain the same, or increase to three minutes incubation time. Subsequently, the slides held in slide trays were immersed for 15 second intervals in a new bath of deionized water three times. For dehydration, slides held in slide trays were immersed for less than 1 second intervals ten times in each of the following solutions: 95% ethanol, 100% ethanol, and a repeat immersion in a new bath of 100% ethanol for 10 additional dips of less than 1 second each. Slides were evaluated briefly for evidence of excess fading wherein the background tissues turn from very dark blue to very light blue and/or mast cells granules change from red or red/violet to vague dark blue. If excess fading occurs on a test slide, incubation time is adjusted.

**Supplemental data Figure S1**


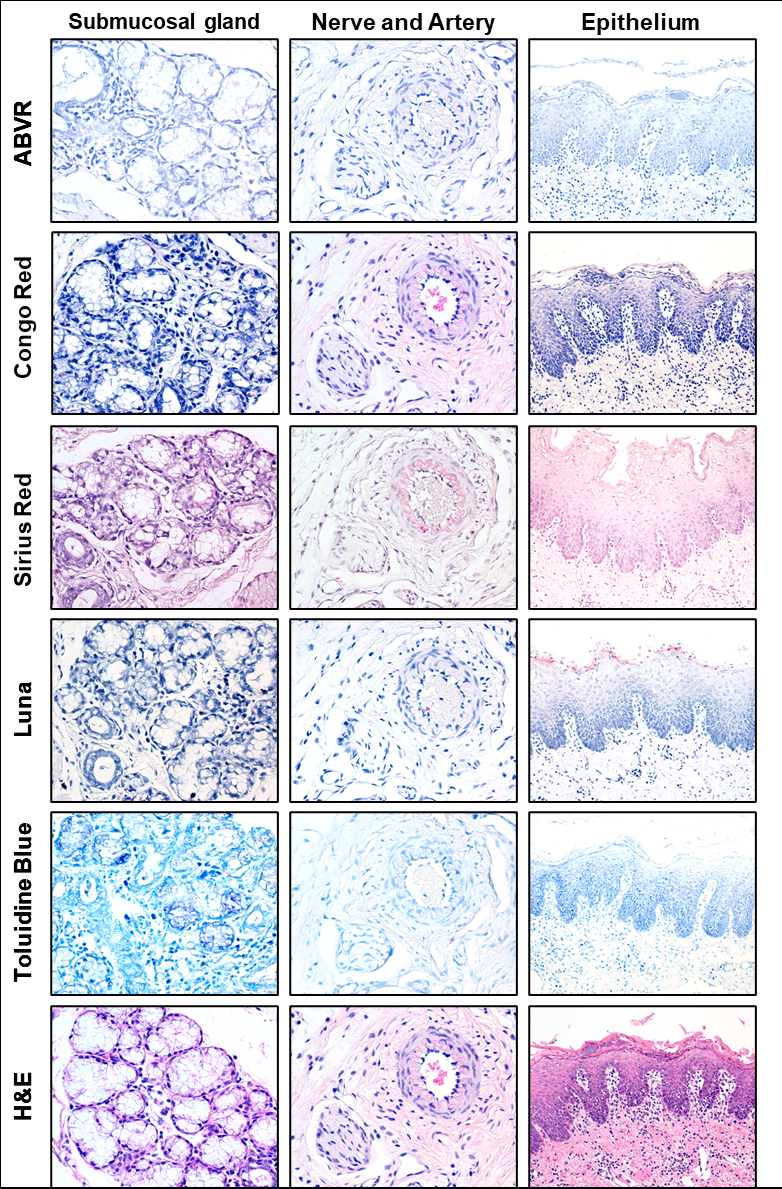


**Supplementary Figure 1**. Stain uptake by surrounding cells and tissues decrease ease of detection. Astra Blue – New Vital Red (ABVR), Sirius Red, Congo Red, Luna, Toluidine blue, and modified regressive hematoxylin and eosin (H&E) were used to stain esophageal tissues.

**Supplementary References**

1. Gill GW. H&E Stain. In Cytopreparation 2013 (pp. 207-216). Springer, New York, NY.
2. Meyerholz DK, Griffin MA, Castilow EM, Varga SM. Comparison of histochemical methods for murine eosinophil detection in an RSV vaccine-enhanced inflammation model. Toxicol Pathol. 2009 Feb;37(2):249-55.
3. Kiernan JA. Dyes and other colorants in microtechnique and biomedical research. Color Technol. 2006 Feb;122(1):1-21.
4. Duffy JP, Smith PJ, Crocker J, Matthews HR. Combined staining method for the demonstration of tissue eosinophils and mast cells. J Histotechnol. 1993 Jun 1;16(2):143-4.
5. Friend DS, Gurish MF, Austen KF, Hunt J, Stevens RL. Senescent jejunal mast cells and eosinophils in the mouse preferentially translocate to the spleen and draining lymph node, respectively, during the recovery phase of helminth infection. J Immunol. 2000 Jul 1;165(1):344-52.
6. Llewellyn BD. An improved Sirius red method for amyloid. J Med Lab Technol. 1970 Jul;27(3):308.
7. Luna LG. Histopathologic methods and color atlas of special stains and tissue artifacts. American Histolabs, Gaithersburg, MD. 1992.
8. Luna LG. Manual of histologic staining methods of the armed forces institute of pathology. 3rd Ed. New York: McGraw-Hill; 1968. pp. 111–112
9. Hirasawa M, Ito Y, Shibata MA, Otsuki Y. Mechanism of inflammation in murine eosinophilic myocarditis produced by adoptive transfer with ovalbumin challenge. Int Arch Allergy Imm. 2007;142(1):28-39.
